# Human Th1 Cells That Express CD300a Are Polyfunctional and After Stimulation Up-Regulate the T-Box Transcription Factor Eomesodermin

**DOI:** 10.1371/journal.pone.0010636

**Published:** 2010-05-13

**Authors:** Sriram Narayanan, Rodolfo Silva, Giovanna Peruzzi, Yelina Alvarez, Venkateswara R. Simhadri, Karen Debell, John E. Coligan, Francisco Borrego

**Affiliations:** 1 Receptor Cell Biology Section, Laboratory of Immunogenetics, National Institute of Allergy and Infectious Diseases, National Institutes of Health, Rockville, Maryland, United States of America; 2 Laboratory of Molecular and Developmental Immunology, Division of Monoclonal Antibodies, Center for Drug Evaluation and Research, Food and Drug Administration, Bethesda, Maryland, United States of America; New York University, United States of America

## Abstract

Human naïve CD4 T cells express low levels of the immunomodulatory receptor CD300a, whereas effector/memory CD4 cells can be either CD300a^+^ or CD300a^−^. This suggested that CD300a expression could define a specific subset within the effector/memory CD4 T cell subpopulations. In fact, *ex vivo* analysis of the IFN-γ producing CD4 T cells showed that they are enriched in the CD300a^+^ subset. Moreover, stimulated CD4 T cells producing TNF-α and IL-2 besides IFN-γ (polyfunctional) are predominantly CD300a^+^. In addition to producing markedly higher levels of Th1-associated cytokines, the stimulated CD300a^+^ CD4 T cells are distinguished by a striking up-regulation of the T-box transcription factor eomesodermin (Eomes), whereas T-bet is up-regulated in both CD300a^+^ and CD300a^−^ activated CD4 T cells to similar levels. The pleiotropic cytokine TGF-β1 has a determinant role in dictating the development of this Th1 subset, as its presence inhibits the expression of CD300a and down-regulates the expression of Eomes and IFN-γ. We conclude that CD300a^+^ human Th1 cells tend to be polyfunctional and after stimulation up-regulate Eomes.

## Introduction

Upon encountering antigen in secondary lymphoid organs, naïve CD4 T cells differentiate into at least four functionally distinct subsets: Th1, Th2, Th17 and induced regulatory T (iTreg) cells [1]. Th1 cells make IFN-γ and confer immunity against intracellular pathogens [1], [2]. Th1 cells are also involved in the pathogenesis and maintenance of certain autoimmune conditions [1], [3]–[4]. Th2 cells produce IL-4, IL-5 and IL-13 and mediate the response against extracellular parasites and they are involved in the induction of allergic diseases and asthma [5]. Th17 cells make IL-17a, IL-17f, IL-21 and IL-22, mediate immune responses against fungi and extracellular bacteria [1], [6]–[7], and have a role in the pathogenesis of some autoimmune diseases [8]. iTregs produce TGF-β1, IL-10 and IL-35, and play a critical role in maintaining self-tolerance and regulating immune responses [1], [9]–[12].

The cytokines and transcription factors that regulate the fate commitment of CD4 cells have been the subject of very intense investigation. Th1 differentiation is promoted by IL-12 and IFN-γ [1], [13]. These cytokines, together with TCR mediated signals, are very important for the expression of the key fate-determining or master transcription factor of Th1 cells T-bet, a member of the T-box transcription factor family [1], [13]–[14]. Beside T-bet, other lineage specific genes are expressed by Th1 cells. For example, T-bet induces IL-12Rβ2 expression by differentiating Th1 cells [15]. Then, these differentiating Th1 cells can be selected and expanded by IL-12 produced by APCs [16]. Runx3 is another transcription factor that cooperates with T-bet for maximal production of IFN-γ and silencing the gene encoding IL-4 in Th1 cells [17]. Other transcription factors important in Th1 development are STAT-1, the major transducer of IFN-γ signaling, which plays a critical role in the IFN-γ mediated induction of T-bet [18], and STAT-4, the IL-12 signal transducer that is important for the amplification of the Th1 response [19]–[20]. Along with these two STAT proteins, eomesodermin (Eomes), another T-box transcription factor that is critical for IFN-γ production by CD8 T cells [21]–[23], has been suggested to have a role in IFN-γ production by murine CD4 T cells [24]–[25].

The simultaneous measurement of cell surface receptors and intracellular cytokines allows distinction among T cell subsets, particularly in humans [26]. For instance, CCR5 and CXCR3 expression is associated with Th1 cells [27]–[28]. CD4 T cell subsets also express different cytokine receptors that play crucial roles both in their development and phenotypic maintenance. For example, Th1 cells express high levels of IL-12Rβ2 and IL-18Rα [15], [29]–[31]. The expression of surface receptors and intracellular cytokines by each T cell subset likely reflects their distinct functional roles. It should be noted that the general correlation of cell surface receptor expression and cytokine production with particular T cell subsets is not exact [26].

The T cells can also be divided into subsets by the type and number of cytokines that they produce. T cells that produce multiple cytokines simultaneously are commonly referred to as polyfunctional [32]. Several publications have shown that a higher number of polyfunctional T cells is correlated with a better prognosis during HIV infection and vaccine animals studies have shown that the quality of the response, i.e. polyfunctionality, is predictive of control of the infection following challenge [32]–[36].

Here, we report that the expression of the immunomodulatory receptor CD300a defines two subsets of circulating human IFN-γ producing CD4 T cells. *In vitro* TCR stimulation of the CD300a^+^ population led to marked stimulation of Th1 cytokine production with polyfunctionality also correlating with CD300a expression. Such stimulation also led to a striking up-regulation of Eomes expression when compared with the CD300a^−^ subset, whereas T-bet up-regulation does not distinguish the CD300a^+^ and CD300a^−^ subsets. The pleiotropic cytokine TGF-β1 has a determinant role in dictating the development of this Th1 subset, as its presence inhibits the expression of CD300a and down-regulates the expression of Eomes and IFN-γ.

## Results

### Human circulating IFN-γ producing CD4 T cells are predominantly CD300a^+^

**Figure 1:**
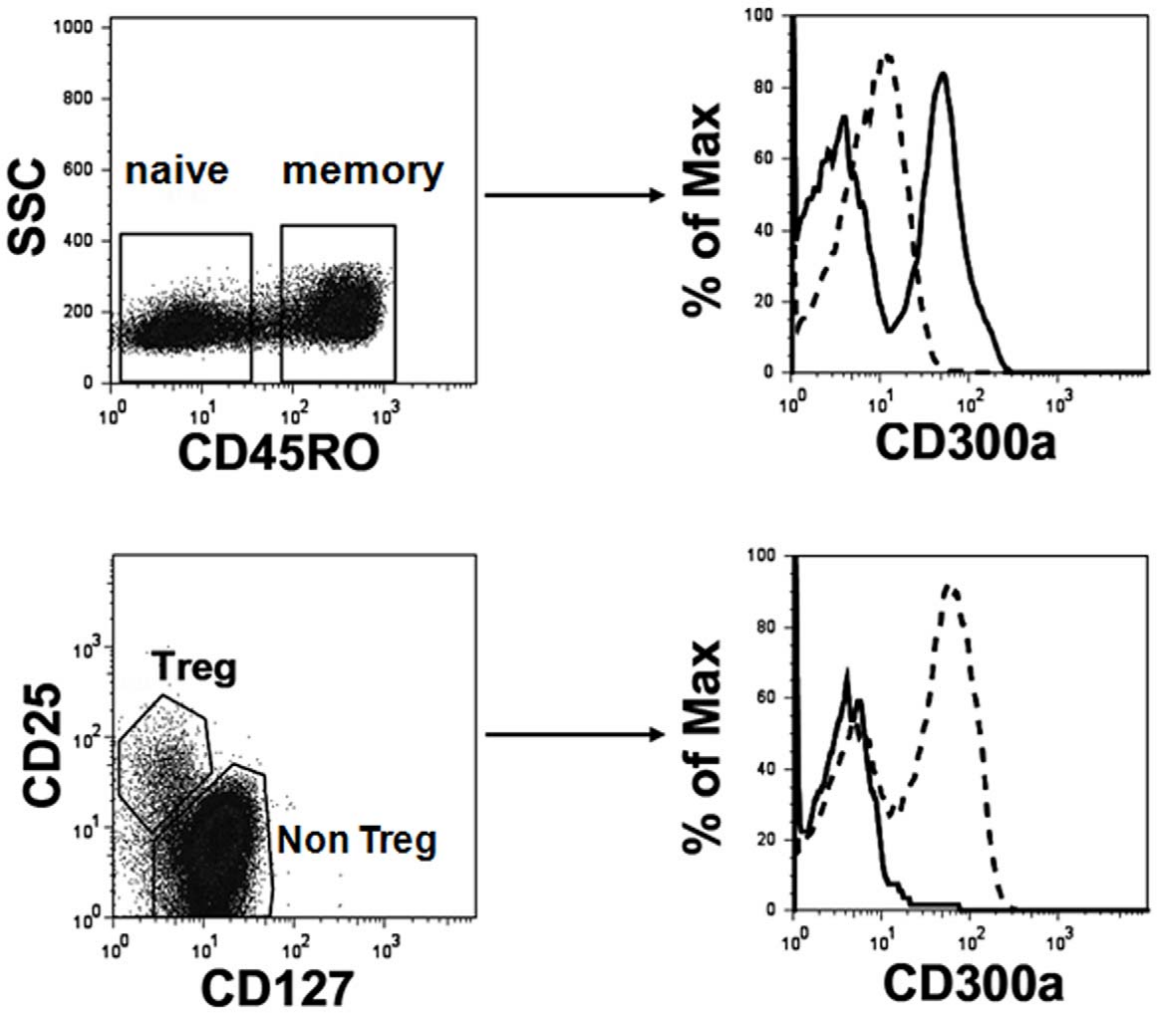
Flow cytometric analysis of human peripheral blood CD4 T cells. In the upper panel, freshly isolated CD4 T cells were labeled with anti-CD4, -CD45RO, and -CD300a mAb. The lymphocyte gate was determined according to the forward and side scatter parameters. Then CD45RO expression was determined for the CD4^+^ cells. The expression of CD300a was assessed in naïve cells (CD45RO^−^, dashed line) and memory cells (CD45RO^+^, continuous line). In the lower panel, freshly isolated CD4 T cells were labeled with anti-CD4, -CD25, -CD127 and -CD300a mAb. After electronically gating the CD4^+^ cells, the expression of CD300a was assessed for the Treg cells (CD25^+^CD127^low^, continuous line) and non Treg cells (dashed line). Results are representative of 12 healthy donors.

The immunomodulatory receptor CD300a is expressed on cells of the myeloid and lymphoid lineages [37]–[43]. Although CD300a is expressed on all NK cells [39], not all T and B lymphocytes express this receptor ([40]–[41] and data not shown), suggesting that CD300a expression by these cells may be a marker of specific T cell subsets. To investigate this, we examined CD300a expression by human CD4 T cell subsets. We found that naïve CD4 T cells, which include the majority of the CD45RO^−^ CD4 T cells from human healthy donors, express low levels of this receptor, whereas the CD45RO^+^ population, which includes the effector and memory cells, is divided into CD300a^+^ and CD300a^−^ populations. Tregs, characterized by the phenotype CD25^+^CD127^low^ [44], are CD300a^−^ (Figure 1).

**Figure 2:**
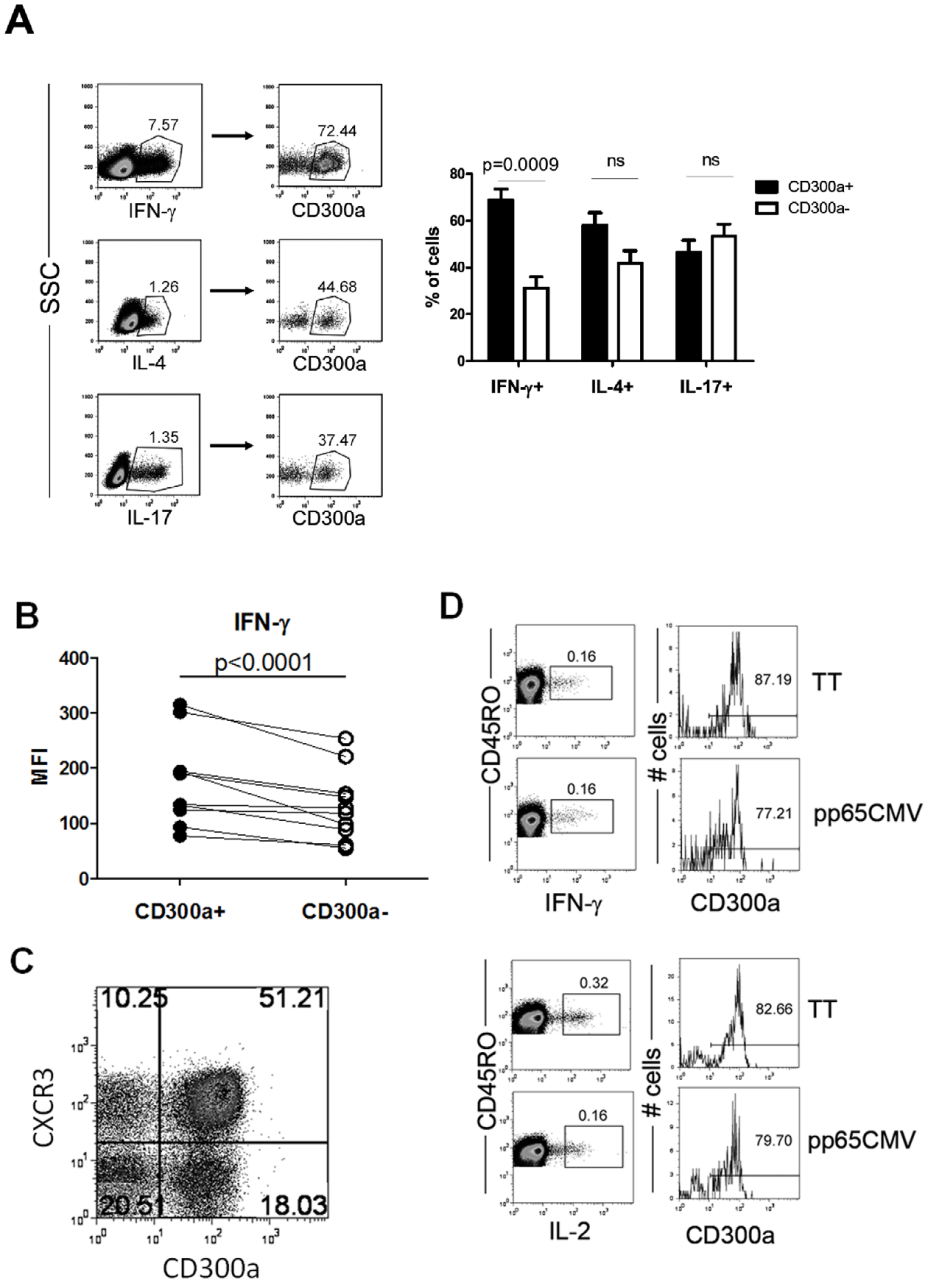
Human circulating IFN-γ producing CD4 T cells are enriched in the CD300a^+^ subset. (A) Flow cytometric analyses for the production of IFN-γ, IL-4 and IL-17 in combination with cell surface expression of CD300a by purified CD4 T cells stimulated with PMA and ionomycin for 4–5 h. The lymphocyte gate was determined according to the forward and side scatter parameters. The CD300a expression was determined for the cytokine producing cells. A representative healthy donor is shown. The bar graph represents the average ± SEM of the percentage of CD300a^+^ cells within each cytokine producing subset. Results are from 15–19 donors. (B) MFI of IFN-γ expression in the cytokine producing memory CD300a^+^ and CD300a^−^ cells. (C) Expression of CXCR3 and CD300a by circulating memory CD4 T cells. Freshly isolated CD4 T cells were labeled with anti-CD4,-CD45RO, -CD300a and -CXCR3. Dot plots are from CD4^+^CD45RO^+^ gated cells. Results from a representative donor are shown from four donors that were analyzed. (D) PBMCs from healthy donors were stimulated overnight with CMV pp65 or TT in the presence of brefeldin A and then analyzed for the production of IFN-γ and IL-2 by memory CD4 T cells (CD4^+^CD45RO^+^). For the cytokine positive cells, the cell surface expression of CD300a was measured. The response of two of the four donors analyzed is shown.

We explored whether CD300a expression could be utilized to further discriminate among Th effector memory subsets. To investigate this, we stimulated purified human CD4 cells with PMA and ionomycin and then categorized them according to their signature cytokine production, i.e. Th1 (IFN-γ), Th2 (IL-4) and Th17 (IL-17). We show that the IFN-γ producing CD4 T cells are predominantly CD300a^+^ (∼70%), whereas both the IL-4 and IL-17 producing CD4 T cells are equally distributed between the CD300a^+^ and CD300a^−^ subsets (Figure 2A). We then checked the level of cytokine production by the stimulated cells by measuring the median fluorescence intensity (MFI) of cytokine staining, a value known to be correlated with the amount of cytokine produced by a T cell [32].We observed that within the IFN-γ producing cells the CD300a^+^ subset produced more IFN-γ per cell than the CD300a^−^ subset (Figure 2B). These results indicate that not only are IFN-γ producing cells enriched in the CD300a^+^ subpopulation, but also, on a per cell basis, the CD300a^+^ cells tend to produce higher levels of IFN-γ than the CD300a^−^ subset. We also checked the expression of the cytokine receptor CXCR3, a chemokine receptor preferentially expressed on Th1 cells [27]–[28], on the CD300a^+^ and CD300a^−^ subsets. Our results show that for CD4^+^CD45RO^+^ cells the CD300a^+^ subset is predominantly CXCR3^+^, confirming that Th1 cells are enriched in the CD300a^+^ subset (Figure 2C). Finally, and in order to further confirm that Th1 cells are enriched in the CD300a^+^ subset, we measured the IFN-γ production by Ag-specific CD4 cells. Healthy donors were analyzed for their response to the antigens pp65 from human cytomegalovirus (CMV pp65) and tetanus toxoid (TT). It is estimated that more than 50–60% of the US population is CMV^+^ and virtually everyone has been vaccinated for tetanus. We analyzed four healthy donors and found that two responded to CMV pp65, and all responded to TT (data not shown). Results shown in Figure 2D indicate that more than 75% of the Ag-specific, IFN-γ producing cells are positive for CD300a. We also measured the production of IL-2 in response to the same antigens and found that more than 75% of the IL-2 producing cells are CD300a^+^ (Figure 2D). Altogether, the above results indicate that the circulating pool of human Th1 cells is predominantly CD300a^+^.

**Figure 3:**
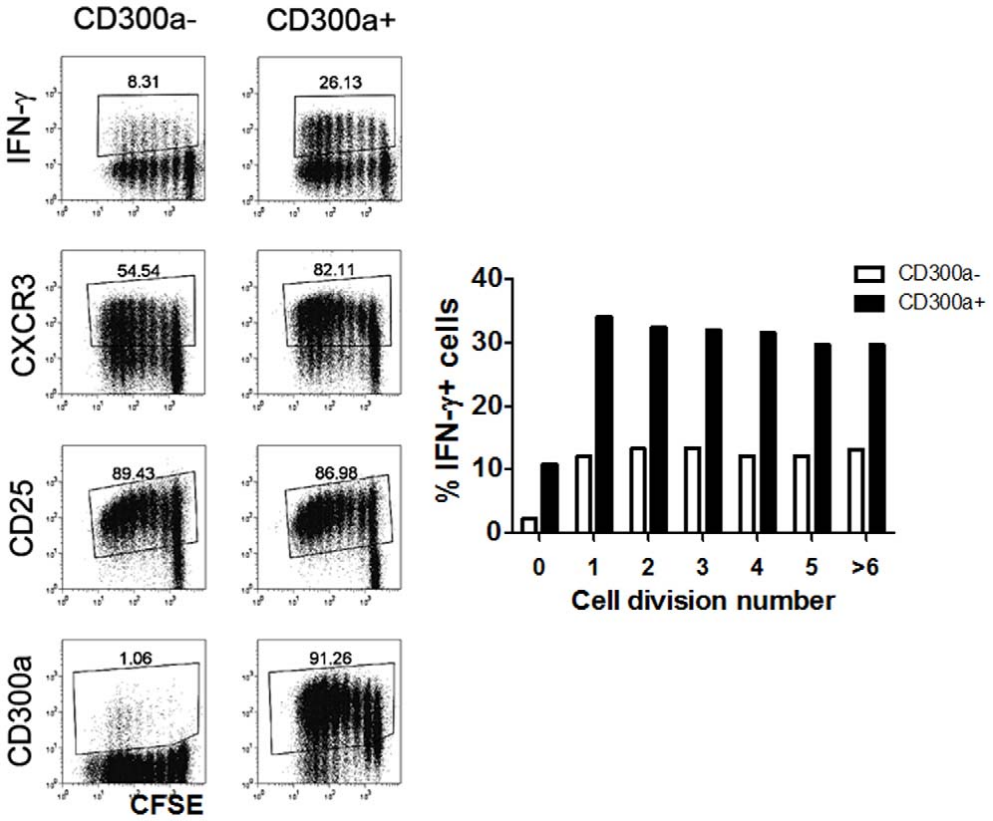
Proliferation and phenotype of CD45RO^+^CD300a^+^ and CD45RO^+^CD300a^−^ CD4 T cells. Proliferation, as measured by CFSE dilution, of sorted CD45RO^+^CD300a^+^ and CD45RO^+^CD300a^−^ cells in response to plate bound anti-CD3 plus anti-CD28 mAb. After three days of stimulation, cells were rested four additional days. After the resting period, the cell surface expression of CXCR3, CD25 and CD300a was measured. IFN-γ production was measured by intracellular staining after a brief stimulation with PMA and ionomycin for 4 h. The bar graph represents the percentage of IFN-γ^+^ cells for each cell generation in the CD300a^+^ and CD300a^−^ subsets. Results shown are representative of four different donors.

As it has been previously shown that cytokine repertoire in Th cells is regulated by cell division [45]–[47], we next considered the possibility that the CD300a^+^ subset may produce more IFN-γ because they proliferate at a higher rate than CD300a^−^ cells. To do this, we sorted CD4^+^CD45RO^+^ cells into CD300a^+^ and CD300a^−^ subsets. We labeled these cells with CFSE and stimulated them with anti-CD3 plus anti-CD28 mAb for 72 h. Then, cells were rested for four days and briefly re-stimulated with PMA plus ionomycin. As shown in Figure 3, although CD300a^−^ cells tend to proliferate only slightly less than the CD300a^+^ cells, the fraction of IFN-γ producing cells within each subset of dividing cells is smaller for the CD300a^−^ cells than in the CD300a^+^ cells supporting our previous observation (see above) that CD300a^−^ cells intrinsically produce less IFN-γ than CD300a^+^ cells. We also observed that more of the proliferating CD300a^+^ cells are CXCR3^+^ than the CD300a^−^ cells confirming the results obtained *ex vivo*. However, the expression of the activation marker CD25 is similar for both the CD300a^+^ and CD300a^−^ subsets, indicating that there is no difference in the activation potential of each subset. We also observed that the presence or absence of CD300a on the cell surface is a permanent feature, namely, that it is maintained throughout cell division under the culture conditions used in these experiments.

### CD300a^+^ Th1 cells are polyfunctional

**Figure 4:**
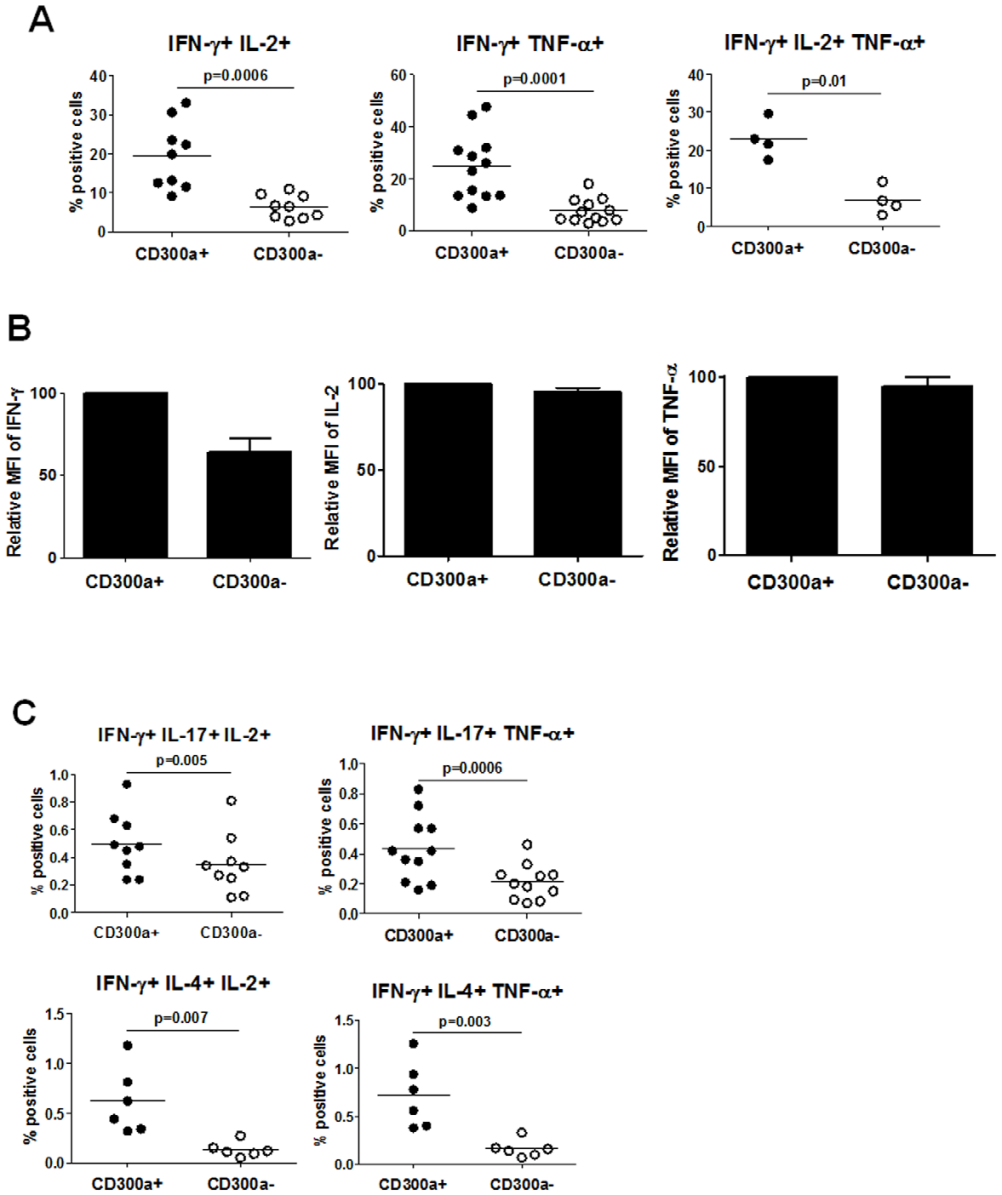
CD300a^+^ CD4 T cells are more polyfunctional than CD300a^−^ CD4 T cells. (A) Purified CD4 T cells were stimulated with PMA and ionomycin for 4–5 h. Then, cells were stained for cell surface expression of CD300a and production of IFN-γ and TNF-α and/or IL-2. The percentage of double and triple producers in the CD300a^+^ and CD300a^−^ cells is shown. Each symbol represents a different donor. (B) Graphic representation of the average ± SEM of the MFI of IFN-γ, IL-2 or TNF-α for the triple producers shown in panel A. (C) Purified CD4 T cells were stimulated with PMA and ionomycin for 4–5 h. Then, cells were stained for cell surface expression of CD300a and the production of the indicated cytokines. The percentage of triple cytokine producers in the CD300a^+^ and CD300a^−^ subsets is shown. Each symbol is a different donor.

Others have shown that there are differences in the quality of effector cells based on whether they secrete multiple cytokines [32]–[36]. The generation of Ag-specific polyfunctional CD4 and CD8 T cells during vaccination regimes has been directly correlated with protection against subsequent parasite or viral challenge [35]–[36]. Moreover, the number of Ag-specific polyfunctional T cells is strongly correlated with the outcome of certain infectious diseases [32]. Given that CD300a^+^ cells produce more IFN-γ per cell (see Figure 2B) than the CD300a^−^ cells, we wanted to determine if the expression of CD300a correlates with polyfunctionality. First, we analyzed for CD4 T cells producing combinations of IFN-γ, TNF-α and IL-2. We found that cells that produce two cytokines (IFN-γ and IL-2 or IFN-γ and TNF-α) or three cytokines (IFN-γ, IL-2 and TNF-α) simultaneously (polyfunctional) are markedly enriched in the CD300a^+^ subset (Figure 4A). Of relevance to the correlation of IFN-γ production with CD300a expression, the MFI for IFN-γ staining is much greater for the CD300a^+^ triple producers than for the CD300a^−^ triple producers (Figure 4B). However, the MFI for TNF-α or IL-2 staining is the same in the CD300a^+^ and CD300a^−^ triple producers. Next, cells producing IFN-γ plus TNF-α or IL-2 in combination with IL-4 or IL-17 were enumerated. Although there are very few triple positive cells for these combinations, results presented in Figure 4C clearly show that polyfunctionality correlates with CD300a expression. Altogether, our results indicate that Th1 cells, as classified by IFN-γ production, contain a population of cells enriched for CD300a expression that are markedly more polyfunctional than the CD300a^−^ cells.

### *Ex vivo* activated CD300a^+^ and CD300a^−^ cells have a very similar Th1 signature, but are distinguishable by cytokine production and Eomes expression

**Figure 5:**
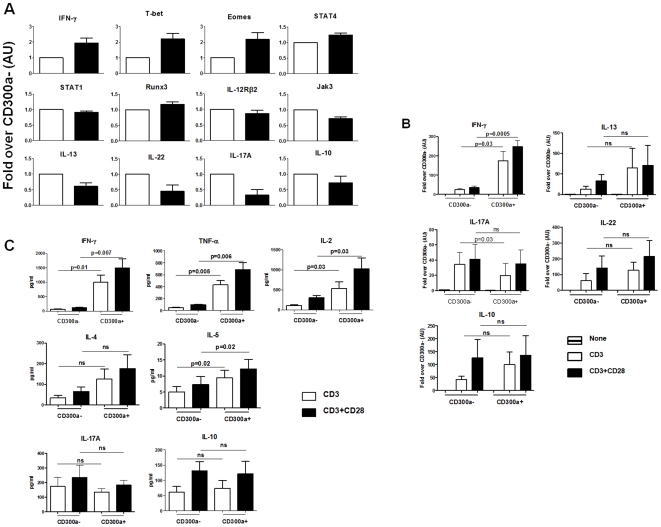
The cytokine and transcription factor signature of CD300a^+^ and CD300a^−^ human memory CD4 T cells. (A) Purified CD4 T cells were sorted into CD45RO^+^CD300a^+^ (black bars) and CD45RO^+^CD300a^−^ (white bars). RNA was extracted and the levels of mRNAs were determined by real-time PCR. Graphs represent the average ± SEM. AU: arbitrary units. Data are from 5–6 donors. These data correspond to the untreated (none) samples in Figures 5B and 6A. (B) Purified CD4 T cells were sorted into CD45RO^+^CD300a^+^ and CD45RO^+^CD300a^−^ and then stimulated overnight with plate bound anti-CD3 or anti-CD3 plus anti-CD28 mAb. RNA was extracted from cells and specific cytokine mRNAs were quantified by real-time PCR. (C) Supernatants from the cultures were harvested and the levels of cytokines were measured.

The above results indicate that human circulating Th1 cells are enriched for CD300a expression. To further explore the molecular signature associated with the CD300a^+^ phenotype, we sorted peripheral blood CD4^+^CD45RO^+^ cells into CD300a^+^ and CD300a^−^ cells and extracted the RNA for analysis of cytokine and transcription factor transcripts involved in Th1 development. We observed (Figure 5A) that freshly isolated, unstimulated CD300a^+^ cells express approximately twice as much IFN-γ, T-bet and Eomes mRNA, and significantly more STAT-4 mRNA than the CD300a^−^ subset. There were no significant differences between the two cell subsets in the IL-12Rβ2, STAT-1 or Runx3 mRNA levels. Both IL-12Rβ2 and Runx3 are known to be involved in Th1 development downstream of T-bet [15], [17]. Jak3 has also been implicated in Th1 development [48], and we observed that the Jak3 mRNA levels are slightly decreased in the CD300a^+^ subset compared to the CD300a^−^ subset. Overall, these results support the view that Th1 cells are enriched in the CD300a^+^ subset. On the other hand, the IL-13, IL-17A and IL-22 mRNA levels are significantly decreased in the CD300a^+^ subset compared to the CD300a^−^ subset, indicating that Th2 and Th17 cells are not enriched for CD300a expression.

The sorted CD300a^−^ and CD300a^+^ populations were stimulated overnight with anti-CD3 or anti-CD3 plus anti-CD28 mAb. Then, we measured cytokine mRNA (Figure 5B) and cytokine levels in the culture supernatant (Figure 5C). The results clearly show that production of the Th1 signature cytokine, IFN-γ, and other cytokines associated with Th1 cells, TNF-α and IL-2, are dramatically higher for the TCR activated CD300a^+^ cells. This agrees with the results reported above for cells stimulated with PMA and ionomycin. No other cytokines show a significant difference in their levels of production.

**Figure 6:**
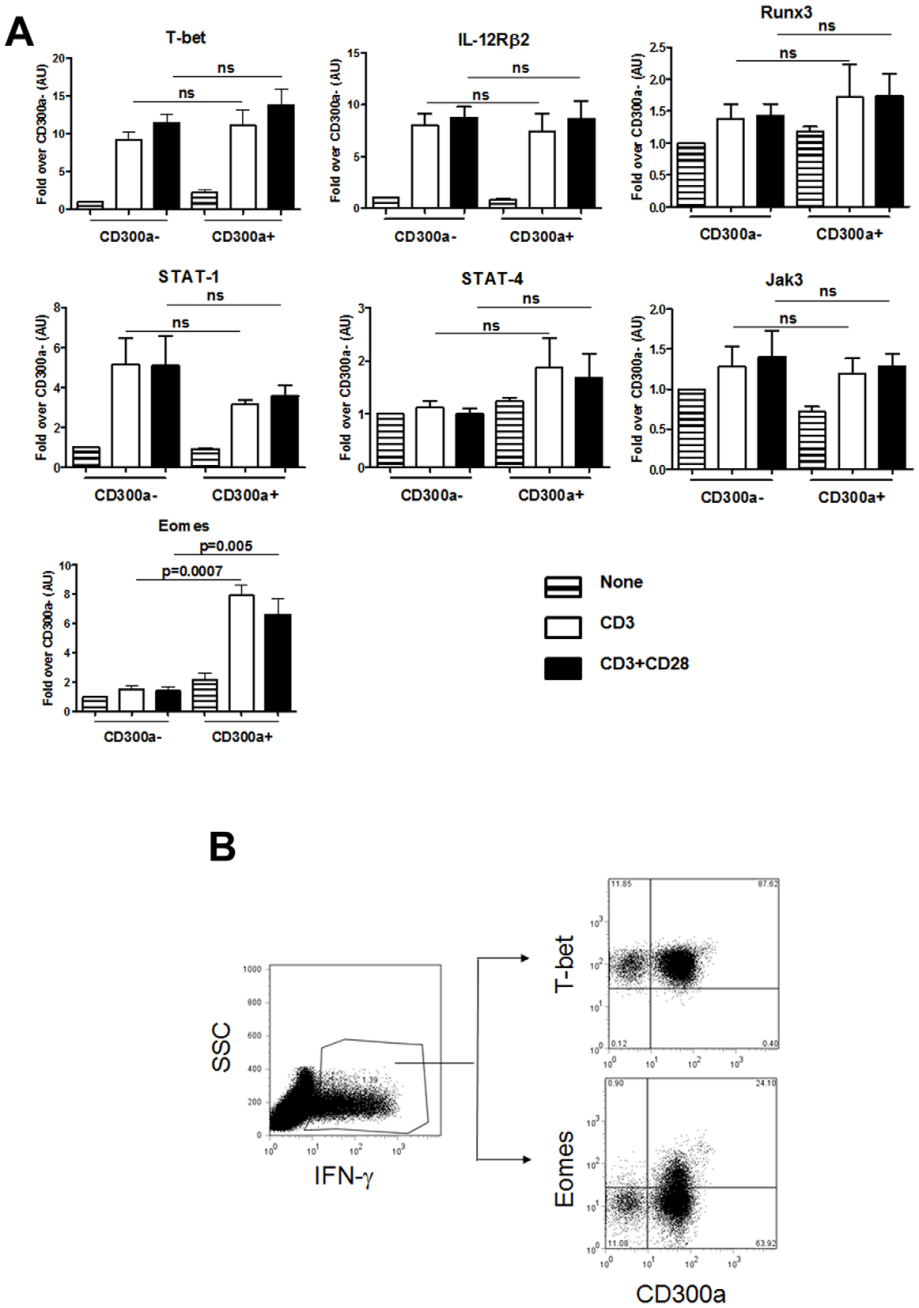
CD300a^+^ cells up-regulate Eomes after stimulation. (A) mRNAs for the indicated transcription factors, and IL-12Rβ2 and Jak3 were quantified by real-time PCR. Graph represents the average ± SEM. AU: arbitrary units. Data are from 5–6 donors. (B) Purified CD4 T cells were stimulated with plate bound anti-CD3 plus anti-CD28 mAb for 24 h. The lymphocyte gate was determined according to the forward and side scatter parameters. Then cells were stained for cell surface expression of CD300a and for the intracellular expression of IFN-γ. The expression of Eomes, T-bet and CD300a was determined in the IFN-γ^+^ cells. Results are representative of three donors.

Because TCR stimulation shows that CD300a expression strongly correlates with a higher production of IFN-γ, we suspected that the transcription factors important for Th1 development, namely T-bet, Eomes, Runx3, STAT-1 and STAT-4, as well as other Th1 associated proteins like Jak3 and IL-12Rβ2, would be differentially expressed by activated CD300a^+^ and CD300a^−^ cells. We were surprised to find that TCR stimulation increased the expression of T-bet mRNA, the master transcription factor for Th1 development, to the same degree in both CD300a^+^ and CD300a^−^ cells (Figure 6A). Consequently, the induced expression levels of IL-12Rβ2 mRNA were the same, as *IL-12Rβ2* is a target gene of T-bet [15]. None of the other mRNAs measured showed a significant difference in the levels of expression between CD300a^+^ and CD300a^−^ cells except for the T-box transcription factor, Eomes. We observed a dramatic increase in Eomes mRNA expression in the CD300a^+^ subset after TCR stimulation relative to the CD300a^−^ subset. Then, we determined by flow cytometric analysis if the activated CD300a^+^ subset has higher levels of Eomes protein than the activated CD300a^−^ subset. To do that, purified CD4 T cells were stimulated with anti-CD3 plus anti-CD28 mAb and the IFN-γ producing cells were identified by intracellular staining. Results in Figure 6B show that T-bet was equally expressed by both the CD300a^+^ and CD300a^−^ IFN-γ producing cells, while Eomes was expressed at higher levels by the CD300a^+^ subset. Altogether, these results show that the CD300a^+^ and CD300a^−^ cells have a very similar Th1 signature except, after stimulation, the CD300a^+^ cells up-regulate Eomes expression and tend to produce significantly larger amounts of IFN-γ, often in association with TNF-α and/or IL-2.

### Th1 polarization in the presence of TGF-β1 induces down-regulation of IFN-γ and Eomes, but not T-bet expression

**Figure 7:**
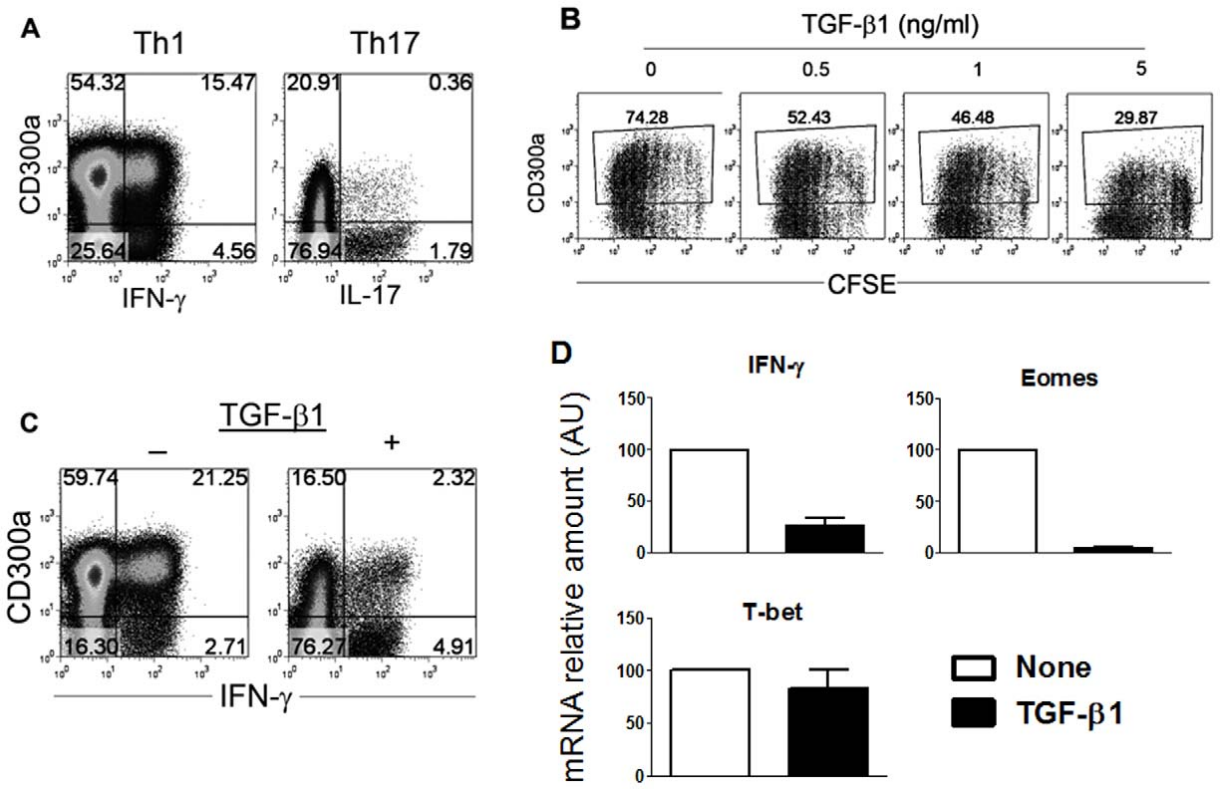
TGF-β1 down-regulates the expression of CD300a and Eomes in Th1 cells. (A) Sorted naïve CD4 T cells were polarized under Th1 or Th17 conditions as described in materials and methods. At day 12, cells were stimulated with PMA and ionomycin for 4 h, after which the cell surface expression of CD300a and the production of IFN-γ or IL-17 was determined. Results from a representative donor are shown from four donors that were analyzed. (B) Proliferation, as measured by CFSE dilution, of purified CD4 cells in response to plate bound anti-CD3 plus anti-CD28 mAb in the absence or presence of increasing amounts of TGF-β1. After three days of stimulation cells were rested four additional days. After the resting period, the cell surface expression of CD300a was measured. Results from a representative donor are shown from three donors that were analyzed. (C) Sorted naïve CD4 T cells were polarized under Th1 conditions in the absence or presence of 5 ng/ml of TGF-β1. At day 12, cells were stimulated with PMA and ionomycin for 4 h and the cell surface expression of CD300a and production of IFN-γ were determined. Results from a representative donor are shown from four donors that were analyzed. (D) Sorted naïve CD4 T cells were polarized under Th1 conditions in the absence or presence of 5 ng/ml of TGF-β1. At day 12, cells were stimulated with plate bound anti-CD3 + anti-CD28 mAb overnight, followed by RNA extraction. Specific mRNAs for IFN-γ, T-bet and Eomes were quantified by real-time PCR. Bar graphs show the averages of the percentage decrease in the presence of TGF-β1 ± SEM. Data are from 4 donors.

Our results indicate that CD300a expression distinguishes a major human memory CD4 Th1 cell population that upon TCR stimulation produces relatively high levels of IFN-γ, often in association with other cytokines (polyfunctional capability), and markedly up-regulates the expression of Eomes. In order to determine conditions that promote the development of these CD300a^+^ cells, we sorted naïve CD4 cells from human peripheral blood and cultured them in different conditions. Cells cultured under Th1 polarizing conditions are mostly CD300a^+^ and this population included most of the IFN-γ producing cells (Figure 7A). On the other hand, cells cultured under Th17 polarizing conditions were mostly CD300a^−^ and this population included most of the IL-17 producing cells (Figure 7A). These *in vitro* experiments are in agreement with the results obtained *ex vivo*, where circulating memory Th1 cells are enriched for CD300a expression and Th17 cells tend to be CD300a^−^ (see Figure 2A).

Using the *in vitro* differentiation assay, we sought to determine what signals control the expression of CD300a, Eomes and IFN-γ production on effector/memory cells. TGF-β1 was an obvious candidate to study because both Th17 cells and Treg cells require this cytokine for their development, and our results show that Treg cells are CD300a^−^ and Th17 cells tend to be enriched in the CD300a^−^ subset. In fact, when naïve CD4 T cells are polarized under Th17 conditions in the absence of TGF-β1, in addition to generating less IL-17 producing cells, the number of CD300a^−^ cells decreased compared to cultures where TGF-β1 was present (data not shown). Therefore, we analyzed the ability of TGF-β1 to modulate the expression of CD300a by total CD4 cells. In Figure 7B, we show that addition of TGF-β1 to cell cultures results in a decreased number of cells expressing CD300a. This decrease in the number of CD300a^+^ cells correlated with the amount of TGF-β1 present in the culture media. Next, we cultured cells under Th1 polarizing conditions in the presence of TGF-β1. After 12 days in culture, cells were briefly re-stimulated with PMA and ionomycin, and the expression of CD300a and the production of IFN-γ were determined. In Figure 7C, we show that the presence of TGF-β1 during priming and differentiation of Th1 cells induced a dramatic increase in the CD300a^−^ cells. Under these culture conditions, there was also a decrease in the frequency of IFN-γ producing cells and the majority of IFN-γ producing cells were in the CD300a^−^ subset (Figure 7C). Finally, we looked at the expression of T-bet and Eomes by the *in vitro* differentiated Th1 cells in a recall assay in the presence and absence of TGF-β1. Results depicted in Figure 7D showed that the level of IFN-γ mRNA was much lower in cells that were cultured in the presence of TGF-β1, which agrees with the intracellular staining results presented in Figure 7C. We also observed that the levels of T-bet mRNA expression in cells exposed or not to TGF-β1 were relatively similar. On the other hand, the levels of Eomes mRNA were extremely sensitive to the presence of TGF-β1 during the *in vitro* differentiation of Th1 cells. Altogether, these results indicate that TGF-β1 is a key factor in dictating the development of Th1 cells characterized by the absence of CD300a and Eomes expression and by the production of less IFN-γ per cell.

## Discussion

We have identified a subset of human circulating Th1 cells that are characterized by their expression of CD300a, the production of relatively large amounts of IFN-γ on a per cell basis, tendency to be polyfunctional in regard to cytokine production, and up-regulation of the T-box transcription factor Eomes during the recall response. The generation of this subset is negatively regulated by the presence of TGF-β1 during priming and differentiation. Our *in vitro* cultures with CD4 naïve T cells show that Th1 polarization in the absence of TGF-β1 (Figure 7A and 7C) results in the generation of mostly CD300a^+^ cells and the vast majority of the IFN-γ producing cells in these cultures are also CD300a^+^. These *in vitro* data correlate with the results obtained *ex vivo* (Figure 2A) showing that the IFN-γ^+^ cells are predominantly CD300a^+^. However, more variability was found in the CD300a expression by the IFN-γ producing cells obtained *ex vivo*, presumably reflecting the presence of TGF- β1 during the *in vivo* priming and differentiation.

The simultaneous measurement of cell surface markers and intracellular cytokines, particularly in human immunology, is becoming a powerful tool for the identification of specific T cell subsets [26]. For example, functionally distinct human CD4 T cells can be identified according to the expression of chemokine receptors [27]–[28]. However, the extent of this correlation between CD4 T cell subsets and specific surface markers and/or cytokine production is far from perfect [26]. T cell subsets can be further distinguished by whether or not they produce multiple cytokines. T cells capable of producing multiple cytokines have been termed polyfunctional [32]. The fact that some CD4 cells are able to produce more than one Th signature cytokine at once, for example IFN-γ and IL-17 together, suggests that the currently defined Th populations may be dividable into subpopulations. In line with this, we show that Th1 (IFN-γ producing cells), Th2 (IL-4 producing cells) and Th17 (IL-17 producing cells) subsets can be clearly divided into at least two subpopulations according to their expression of the CD300a receptor. Nonetheless, like certain chemokine receptors that are more associated with specific Th subsets, we also show that Th1 cells are mostly, but not entirely CD300a^+^.

The major cytokines driving Th1 differentiation are IFN-γ and IL-12 [1], [13], which respectively activate the transcription factors STAT-1 and STAT-4. The expression of the master transcription factor for Th1 differentiation, T-bet, is up-regulated by both IFN-γ and IL-12 in the presence of TCR mediated signals [13], [18]. A “two loop model” has recently been proposed in which initial TCR mediated signals plus IFN-γ induce T-bet expression. Then, after termination of the TCR signal, IL-12Rβ2 expression is up-regulated allowing IL-12 to induce a second wave of T-bet expression [13]. This second wave of T-bet, along with the up-regulation of other transcription factors such as Runx3, is required for imprinting Th1 cells for IFN-γ re-expression later on in the recall response [13]. Our data show that the expression of T-bet is up-regulated to similar levels in memory CD4 CD300a^+^ and CD300a^−^ cells after TCR stimulation, indicating that other transcription factors are involved in the differential production of IFN-γ by these two subsets during the recall response. Our results suggest that Eomes is at least one of these factors if not the sole responsible factor. Although the requirement for Eomes has been primarily studied in the development of CTLs [21]–[23], recent publications have shown that Eomes may also have an important role in the development of mouse Th1 cells [24]–[25]. It has also been shown that *Eomes* is epigenetically modified to a state that is associated with gene activation in murine Th1 cells [49].

Our data suggest that there is a relationship between CD300a and Eomes expression. Th1 cells are mostly CD300a^+^ and up-regulate Eomes, while Th17 cells are mostly CD300a^−^ (our data) and do not up-regulate Eomes [49]. This relationship is further supported by the finding that murine Th17 cells have lower levels of Eomes than naïve CD4 cells and that in Th17 cells *Eomes* is epigenetically modified to a state that is associated with gene repression [49]. In line with this, T-bet and Eomes double KO mice have been shown to develop an anomalous CD8 T cell response during viral infections that is characterized by the production of large amounts of IL-17 and minimal amounts of IFN-γ by the virus specific CTLs [22].

TGF-β1 is known to potently inhibit Th1 development [50]. It is not clear how TGF-β1 inhibits IFN-γ expression [51]–[53]. Some authors have proposed that TGF-β1 inhibits Th1 development through down-regulation of T-bet [51]. Others have said that TGF-β1 uses different mechanisms for controlling IFN-γ expression during the priming and recall responses, with the differential involvement of STAT-4 and T-bet [52]. Studies by Yang et al [25] in mice have shown that T-bet independent IFN-γ production and Th1 development are very susceptible to suppression by IL-6 plus TGF-β1. These two cytokines are very important cytokines for mouse Th17 development. They showed that IL-6 plus TGF-β1 inhibited Eomes expression and that over-expression of Eomes is able to inhibit the ability of these cytokines to down-regulate T-bet independent IFN-γ production [25]. Our results show that in humans, the presence of TGF-β1, during priming and differentiation, favors the development of Th1 cells that are characterized by their ability to up-regulate T-bet during a recall assay to levels that are similar to Th1 cells generated in the absence of TGF-β1, whereas TCR induced expression of Eomes in the recall assay is dramatically decreased if Th1 cells are generated in the presence of TGF-β1. Thus, it appears that TGF-β1 is able to suppress the development of the CD300a^+^ Th1 subset that up-regulates Eomes during the recall response.

Several publications have shown that a higher number of polyfunctional T cells correlates with a better prognosis during HIV infection [32]–[34], [54]. Also, vaccine studies with animals have shown that the higher the level of multifunctional T cells the better the control of the infection following challenge [35]–[36]. From our studies, we conclude that CD300a expression is a marker for human memory CD4 T cell polyfunctionality. However, we observed that CD300a^+^ and CD300a^−^ IFN-γ producing cells are equally polyfunctional if both IL-2 and TNF-α are co-produced, but consistent with all of our findings, the production of IFN-γ is much lower on a per cell basis for the CD300a^−^ cells. Furthermore, the CD300a^−^ population fails to up-regulate Eomes after TCR stimulation and has fewer numbers of IFN-γ^+^IL-17^+^ and IFN-γ^+^IL-4^+^ double producing cells.

Regulation of immune responses by inhibitory receptors is crucial for proper function of the immune system [55]. ITIM(s) containing receptors control a vast array of cellular responses, ranging from autoimmunity, graft versus host disease, allergy, to cell death [55]. The role of CD300a inhibitory receptor during the immune response and more specifically on T cell function remains to be established. Our data show that the differential expression of CD300a clearly distinguishes between two IFN-γ producing Th1 subsets. An important point to consider is that, if CD300a^+^ cells encounter their antigen and the currently unknown CD300a ligand at the same time, their TCR mediated signals would be potentially down-modulated (data not shown). This suggests that after TCR stimulation the polyfunctional Th1 cells, i.e. the CD300a^+^ Th1 cells, may need additional controls in order to avoid Th1 mediated immunopathology.

In conclusion, we propose that there are at least two types of human CD4 IFN-γ producing cells that are distinguishable by the expression of CD300a, and that TGF-β1 exposure favors the development of the CD300a^−^ subset. More studies are required to delineate the exact roles that these two subsets play in T cell mediated immunity.

## Materials and Methods

### Reagents

Antibodies and reagents used in this study were obtained from the following vendors: purified and PE-Cy7 anti-CD3 (clone UCHT1), PE-Cy7 and APC anti-CD4 (clone RPA-T4), PE-Cy7 anti-CD25 (clone BC96), purified anti-CD28 (clone CD28.2), FITC, PE-Cy7 and APC anti-CD45RO (clone UCHL1), APC anti-CD62L (clone Dreg56), FITC anti-CD127 (clone eBioRDR5), FITC and APC anti-IL-2 (clone MQ1-17H12), FITC anti-IL-4 (clone MP4-25D2), FITC anti-IL-17a (clone eBio84DEC17), PE-Cy7 and APC anti-TNF-α (clone Mab11) from eBioscience, Alexa 647 anti-Eomes (clone WD1928). APC anti-CXCR3 (clone 1C6), PE-Cy7 and purified anti-IFN-γ (clone B27) from BD Biosciences. PE anti-CD300a (clone E59.126) that recognizes a unique epitope in CD300a [56] is from Beckman-Coulter. Purified blocking anti-IL-4 (clone 34019) from R&D Systems. Goat anti-mouse (GAM) IgG From Jackson ImmunoResearch Laboratories. All cytokines were from R&D Systems except recombinant IL-2 that was from the NCI. PMA and Ionomycin were purchased from Sigma. Tetanus Toxoid was from Calbiochem and recombinant CMV pp65 from Miltenyi Biotec. CFSE was purchased from Invitrogen. BD Cytofix/Cytoperm Plus kits with GolgiPlug or GolgiStop were used for intracellular cytokine detection and were purchased from BD Biosciences. The Cytokine Bead Array (CBA) human Th1/Th2 kit was purchased from BD Biosciences and the human IL-17A ELISA from eBioscience.

### Flow cytometry

Flow cytometry experiments were performed with a FACS Calibur (BD Biosciences) and the data were analyzed using the FlowJo software package (Treestar). Lymphocytes were electronically gated based on the forward and side scatter parameters. Depending on the experiment, cells were stained with two to four different labeled mAb. Controls included both unstimulated cells and isotype controls that are not included in the figures for clarity purposes.

### Cell isolation and sorting

Buffy coats were obtained from the NIH blood bank from healthy donors. These studies were approved by the NIH and FDA Ethical Committees. CD4 T cells were isolated by negative selection with kits from Miltenyi Biotec (purity was >93%). Naive CD4 T cells, based on a CD62L^+^CD45RO^−^ phenotype, and memory CD45RO^+^CD300a^+^ and CD45RO^+^CD300a^−^ cells, were isolated at the NIAID/NIH and CDER/FDA sorting facilities. After sorting, cells were resuspended in T cell medium: IMDM medium (Invitrogen) containing 10% human AB serum (Valley Biomedical) and supplemented with GlutaMAX (Invitrogen) and penicillin/streptomycin (BioSource International).

### Cell proliferation, differentiation and activation assays

For activation experiments, purified (>93% of purity) CD4 T cells were stimulated with PMA (50 ng/ml) plus Ionomycin (2 µM) for 4–5 h in the presence of GolgiStop (monensin). Then, cell surface receptor expression and intracellular cytokine staining were determined with the BD Cytofix/Cytoperm Plus kit following the manufacturer instructions. A second set of activation experiments was performed on freshly isolated and sorted CD45RO^+^CD300a^+^ and CD45RO^+^CD300a^−^ memory cells. After sorting, cells were resuspended at 0.5–1×10^6^ cells/ml and seeded on anti-CD3 (1 µg/well) or anti-CD3 (1 µg/well) plus anti-CD28 (5 µg/well) mAb coated wells from a 24 well plate. Cells were cultured overnight and the next day supernatants were collected for cytokine measurement and cells were stored at 4°C in RNALater (Ambion) solution until RNA was isolated. Cytokines in the supernatant were measured with a Cytokine Bead Array (CBA) following manufacturer instructions. IL-17A in the supernatant was measured by ELISA.

For proliferation assays, CFSE labeled CD45RO^+^CD300a^+^ and CD45RO^+^CD300a^−^ memory CD4 T cells (0.5–1×10^6^ cells in 2 ml) were added to 24 well plates coated with anti-CD3 (1 µg/well) plus anti-CD28 (5 µg/well) mAb in the presence of 50–100 U/ml of IL-2. Cells were cultured for three days and then transferred into non coated wells for four additional days. Then, a fraction of the cells were collected and the expression of cell surface markers was analyzed by flow cytometry. The rest of the cells were stimulated with PMA (50 ng/ml) plus Ionomycin (2 µM) for 4–5 h in the presence of GolgiStop (monensin) and then cells were permeabilized and fixed and the production of IFN-γ was detected by intracellular staining.

For Ag specific IFN-γ and IL-2 production, PBMC from healthy donors were resuspended at 3×10^6^ cells/ml. Two ml of cells were added to each well from a 24 well plate. Tetanus toxoid (0.5 µg/ml) or CMV pp65 (3 µl/ml) was added to the wells. After a 2–3 h period, 1 µl/ml of Golgi Plug (Brefeldin A) was added to the cultures and the cells were incubated overnight. Next day, IFN-γ and IL-2 production were detected in the CD4^+^CD45RO^+^ memory cells by intracellular staining.

For differentiation assays, sorted naïve (CD62L^+^CD45RO^−^) CD4 T cells were resuspended in T cell medium at 0.5–1×10^6^/ml. Two ml of cells were added to each well of a 24 well plate coated with anti-CD3 (1 µg/well) plus anti-CD28 (5 µg/well). For Th1 differentiation anti-IL-4 (10 µg/ml) mAb and IL-12 (20 ng/ml) were added to the culture. In some Th1 differentiation assays, TGF-β1 (1–5 ng/ml) was added at the beginning of the culture and at day 7. For Th17 differentiation assays, IL-1β (10 ng/ml), IL-6 (50 ng/ml), TGF-β1 (1–5 ng/ml), anti-IL-4 (10 µg/ml) mAb and anti-IFN-γ (10 µg/ml) mAb were added to the culture. IL-23 (20 ng/ml) was added to the culture at days 3 and 5 and, at day 7, TGF-β1 was added again. IL-2 (20 U/ml) was added to all cultures at day 5. At day 12, cells from Th1 and Th17 cultures were harvested and briefly stimulated with PMA and Ionomycin for 4–5 h in the presence of GolgiStop. Cell surface expression of CD300a and intracellular cytokines were measured by flow cytometric analyses. Other cells from the end of the Th1 and Th17 cultures were added to anti-CD3 (1 µg/well) plus anti-CD28 (5 µg/well) mAb coated wells from a 24 well plate and cultured overnight. Next day cells were harvested and RNA isolated for measurement of IFN-γ, T-bet and Eomes transcripts.

### Real-time PCR

All cell samples for RNA isolation were stored at 4°C in RNALater (Ambion) solution. RNA isolation was carried out according to the manufacturer's protocol using RNAqueous4PCR kit (Ambion). cDNA was made using Qscript™ cDNA synthesis kit from Quanta Biosciences. The real-time PCR was done according to the manufacturer's instructions on Roche LC480 real-time machine using Lightcycler® 480 SYBR green I master supermix (Roche Diagnostics). The primers for real-time PCR measurement for human target genes IL-12Rβ2, STAT1, STAT4, JAK1, RUNX3 and Eomes were bought from SA Biosciences Corporation and the primers for IL-13, IL-22, IL-17A, T-bet, IFN-γ, IL-10 and 18s rRNA were obtained from Qiagen. All reactions were done in triplicates and the average value of triplicates was used for calculating the relative levels of each mRNA species. Relative quantification of the target genes was made using the 2^nd^ derivative maximum method using the Roche Lightcycler software and calculating the fold changes over the 18s rRNA levels. Melting curve analysis was performed at the end of each real-time PCR run to make sure that amplification yields only one specific product.

### Statistical analysis

Quantitative data were analyzed using GraphPad Prism software. The data were plotted as bar graphs or scatter plots, and pair wise comparisons were examined by two-tailed paired Student's *t*-test with 99% of confidence interval. *P*<0.05 was considered significant.
